# Donor Functional Warm Ischemic Time in Donation After Circulatory Death Organ Transplantation: Moving Beyond Arbitrary Thresholds

**DOI:** 10.1097/TP.0000000000005871

**Published:** 2026-08-06

**Authors:** Sophie P. Ciere, Jeroen de Jonge, Frank J.M.F. Dor

**Affiliations:** 1 Division of HPB and Transplant Surgery, Erasmus Medical Center Transplant Institute, Erasmus University Medical Center, Rotterdam, The Netherlands.

Globally, transplant programs have adopted diverse strategies to safely incorporate donation after circulatory death (DCD) donors as a major source of organs.^1^ However, DCD introduces specific challenges related to donor functional warm ischemic time (fWIT), a key determinant of ischemia/reperfusion injury. International guidelines have therefore attempted to define and limit acceptable fWIT intervals,^2-4^ although these remain variable and often organ-specific.

Masiero et al^5^ report that kidney and liver transplantation can achieve survival outcomes comparable with donation after brain death, despite a mandated 20-min “asystolic no-touch” interval. This resulted in a median fWIT of 44 min, exceeding conventional thresholds. Although outcomes were acceptable, prolonged fWIT was associated with increased organ discard and higher rates of primary nonfunction (PNF), particularly in uncontrolled DCD where 81% of grafts experienced total warm ischemia of >60 min.^5^ These findings challenge fixed fWIT cutoffs and reinforce the need for physiology-based assessment in the era of machine perfusion.

## ITALY’S 20-MIN STANDOFF IN PERSPECTIVE

The Italian requirement of a 20-min no-touch interval has accelerated adoption of abdominal normothermic regional perfusion (aNRP) followed by ex situ perfusion strategies. This represents an important system adaptation to regulatory constraints. However, international practice remains heterogeneous. In countries with shorter no-touch periods (eg, 5 min), withdrawal of life-sustaining treatment occurs in the intensive care unit, and additional delays occur during transfer, positioning, and cannulation. Legal restrictions on ante-mortem cannulation further influence fWIT.^2^ These logistical factors substantially contribute to variability in ischemic exposure across programs.

Beyond procedural variability, the agonal phase itself is highly heterogeneous. As a result, centers with shorter no-touch intervals may still experience fWIT values comparable with those in jurisdictions with longer legal standoffs. These observations underline that standoff time alone is an insufficient proxy for ischemic injury risk.

## DEFINING THE START OF FWIT: A CALL FOR CONSENSUS

A major limitation in the field is the lack of harmonization in defining fWIT onset. The British Transplantation Society (BTS) DCD guideline emphasizes that fWIT is a physiological continuum rather than a discrete time point. It defines fWIT onset as sustained systolic blood pressure <50 mm Hg (typically for ≥2 min). Importantly, the BTS guideline stresses that injury depends on both severity and duration of hypoperfusion, and highlights organ-specific variability as well as the increasing role of machine perfusion in expanding donor criteria.^6^ The American Society of Transplant Surgeons defines onset as systolic blood pressure <50 mm Hg and recommends documentation of oxygen saturation <70%.^2^ However, SpO_2_ reliability is questionable in profound hypotension. In a liver transplantation cohort, SpO_2_ < 80% preceded hypotension by approximately 7 min and was the only independent predictor of severe ischemia/reperfusion injury.^7^ The American Society of Transplant Surgeons guideline suggests increased complication risk in DCD liver transplantation after 20–30 min of fWIT,^4^ whereas the International Liver Transplantation Society recommends a 30-min upper limit, with explicit recognition that machine perfusion and graft selection may modify these boundaries.^3^

In critical care, mean arterial pressure  <60 mm Hg is associated with impaired organ perfusion.^8^ Accordingly, the International Liver Transplantation Society defines fWIT onset as mean arterial pressure <60 mm Hg and/or SpO_2_ < 80%.^3^ The BTS guideline similarly prioritizes sustained hypotension as the primary determinant of functional ischemia.^6^ Despite these advances, no universal consensus exists, limiting comparability across studies and reinforcing the persistence of arbitrary thresholds.

This lack of standardization is particularly evident in kidney transplantation, where definitions and thresholds vary considerably. Unlike liver transplantation, there is no universally accepted absolute cutoff for kidney grafts. However, commonly used thresholds include fWIT <30 min as standard risk, 30–45 min as extended-risk but acceptable in selected cases, and >45–60 min as associated with significantly increased risk of delayed graft function and PNF.

Historically, many programs relied on a rigid total WIT (tWIT) limit of 120 min. This is increasingly recognized as overly conservative, particularly when the true functional ischemic interval is short. Law et al demonstrated that even donors with tWIT >2 h frequently had fWIT <30 min and proposed that waiting periods up to 4 h after withdrawal may still be acceptable if functional ischemia remains limited.^9^ This fundamentally challenges the “2-h rule” and reinforces the primacy of physiological over chronological assessment.

Across kidney transplantation, increasing warm ischemia correlates with higher rates of delayed graft function, PNF, and reduced graft survival, as demonstrated in the Maastricht experience and subsequent registry studies. However, contemporary decision-making increasingly integrates multiple parameters beyond time alone, including donor age, AKI status, hemodynamic trajectory, perfusion parameters, machine perfusion metrics, lactate clearance, and biopsy findings. This reflects a broader shift from time-based to multiparameter viability assessment.

A contemporary synthesis for DCD kidney transplantation can therefore be summarized as fWIT <30 min as generally acceptable (standard risk), 30–45 min as increased risk but frequently transplantable with machine perfusion or NRP, and 45–60 min as significantly increased risk (highly selective use). tWIT alone is insufficient as a discard criterion.

## THE ROLE OF MACHINE PERFUSION

The favorable outcomes in the Italian series cannot be separated from the routine use of perfusion technology. All used DCD donors underwent aNRP, and 67%–89% of organs subsequently received ex situ perfusion as well. This is an important caveat when interpreting the results. Machine perfusion—whether in situ or ex situ—allows functional assessment of organs that have sustained a prolonged ischemic insult. Biomarkers such as lactate clearance and bile production allow direct functional assessment, enabling viability-based rather than time-based decision-making.

In the present cohort, 89% of DCD livers underwent additional ex situ perfusion after aNRP.^5^ Whether sequential perfusion strategies are necessary, or whether a single modality suffices when robust viability criteria are applied, remains unresolved. In our experience, livers with fWIT up to 56 min have been successfully transplanted after aNRP alone, suggesting that sequential perfusion may not always be required when strict viability criteria are met (Erasmus MC Transplant Institute, unpublished observation, 2026).

## OTHER ORGANS

These principles extend across organs. Ex vivo lung perfusion enables assessment beyond prolonged fWIT, supporting acceptance based on function rather than time. In kidney transplantation, however, no validated functional viability test exists, and prospective studies are required. Ex vivo heart perfusion similarly allows direct assessment of myocardial performance, rendering the current 30-min fWIT threshold for DCD heart transplantation likely arbitrary.

## FROM EVIDENCE TO IMPLEMENTATION

Translating these advances into clinical practice remains challenging. Although viability assessment expands the donor pool, logistical constraints, including cost, workforce, infrastructure, and time, must be considered. Universal prolonged perfusion of all organs after extended ischemia may not be feasible. The UK experience, with procurement-to-perfusion intervals of up to 4 h, has not resulted in higher utilization compared with shorter pathways,^1,10^ highlighting the need for an upper limit to procurement and assessment time.

A structured implementation framework is therefore required. In the Netherlands, such an approach is being adopted: DCD livers without additional risk factors and fWIT <45 min proceed directly to transplantation after aNRP, whereas fWIT >45 min mandates viability assessment. A forthcoming Dutch randomized controlled trial comparing in situ and ex situ perfusion will evaluate this strategy prospectively.

Finally, viability assessment is not without limits. At very prolonged fWIT, particularly in uncontrolled DCD, the probability of identifying suitable grafts decreases while resource use increases. These boundaries require prospective evaluation using systematic donor-to-outcome tracking, ideally through structured organ utilization pathways such as those proposed by Masiero et al.^5^
